# The Assessment of Dry Eye Syndrome in Relation to the Use of Contact Lenses in the Jazan Region of Saudi Arabia: A Cross-Sectional Study

**DOI:** 10.7759/cureus.72247

**Published:** 2024-10-24

**Authors:** Faisal Abusageah, Essam Alhazmi, Bander Otayf, Taif Zogel, Wafa Alharbi, Faisal Hakami, Ebrahim Abulqusim, Ramis Manni, Hassan Moafa, Ibrahim M Dighriri, Abdulaziz Alagsam

**Affiliations:** 1 Department of Ophthalmology, Prince Mohammed Bin Nasser Hospital, Jazan, SAU; 2 Faculty of Medicine, Jazan University, Jazan, SAU; 3 Facility of Medicine, Jazan University, Jazan, SAU; 4 Department of Otolaryngology, King Fahd Central Hospital, Jazan, SAU; 5 Department of Tropical Medicine, College of Public Health and Tropical Medicine, Jazan University, Jazan, SAU; 6 Department of Pharmacy, King Abdulaziz Specialist Hospital, Taif, SAU

**Keywords:** contact lens, dry eye disease, ocular surface disease, risk factor, saudi arabia

## Abstract

Background

Contact lenses (CL) are frequently used among the population. Various symptoms, including dryness, impaired vision, foreign body sensation, and eye strain, were reported by patients with contact lens-associated dry eye (CLADE) disease. The purpose of this study is to estimate the prevalence and severity of dry eye symptoms among contact lens users, as well as non-contact lens users, and the related variables.

Methods

This descriptive cross-sectional study was conducted among contact lens users, as well as non-contact lens users. The questionnaire was adopted from the literature and translated into Arabic. General and demographic data were collected, and questions about hygiene behaviors, the Contact Lens Dry Eye Questionnaire-8 (CLDEQ-8), and the Ocular Surface Disease Index (OSDI) were answered. IBM SPSS version 27.0.1 (IBM Corp., Armonk, NY) was used for data analysis.

Results

This study involved 367 participants, predominantly women (318, 86.6%) with an average age of 31.0 years. One hundred eighty-one (49.3%) used contact lenses, primarily on a monthly basis. One hundred forty-six (80.7%) of contact lens users reported good hygiene practices. Dry eye symptoms varied in severity, with 139 (37.6%) of the participants experiencing severe dry eye, while many reported symptoms as infrequent or mild. Statistical analysis revealed no significant association between contact lens use and dry eye severity (p = 0.416), but 99 (54.7%) of the participants reported contact lens-associated dry eye (CLADE). Sociodemographic factors showed no significant correlation with contact lens discomfort (CLD) prevalence; however, poor hygiene practices were significantly associated with higher CLD rates (100% versus 52.1%, p = 0.014).

Conclusion

The study found that dry eye symptoms were prevalent among all participants (139, 37.6%) but not necessarily linked to contact lens use. The data indicate that, while many users maintain proper hygiene, a significant number nevertheless have varied degrees of dry eye symptoms, necessitating more inquiry into the mechanisms leading to these concerns. A future study using clinical diagnosis with a tear film breakage test is recommended as a more reliable technique for diagnosing dry eye syndrome (DES).

## Introduction

One of the significant risk factors for dry eye is wearing contact lenses (CL) [1,2]. Discomfort and dryness symptoms are more common and severe in soft contact lens (SCL) wearers, and they intensify more during lens usage than in non-wearers [3,4].

Of contact lens wearers, 10%-50% discontinue their use after three years of starting, with contact lens discomfort (CLD) [5] being the most prevalent cause, with 70% experiencing CLD late in the day [6]. Forty percent of the soft contact lens wearers reported dry eye as the most common reported complaint. However, 25% experience moderate to severe symptoms of dry eye syndrome (DES) [7-9], leading to reduced wearing times [10].

Dry eye syndrome is a multifactorial, chronic condition. It is a tear film condition carried on by increased tear evaporation or a tear deficit that damages the interpalpebral ocular surface and is linked to sensations of ocular discomfort [11]. A lack of any one or more of the tear film's constituents can result in dry eye. Dry eye problems can get worse by things such as using contact lenses and being in unfavorable environmental conditions. A new study has revealed additional risk factors, such as age, contact lens wear, injury to the lacrimal and eyelid glands, and autoimmune processes [12].

The symptoms of dry eye include eye redness and discomfort, causing burning sensations, foreign body sensation, and dry eyes. In more advanced stages, ocular discharges may cause hazy vision, which over time may progressively degrade visual acuity. The Saudi population is in danger of developing dry eye syndrome (DES) due to a number of environmental and epidemiological risk factors [13], as well as an increased use of contact lenses, particularly among female medical students [14]. Saudi Arabia's scorching desert environment, with summer temperatures reaching 50°C, is common across the country [13].

Dry eye illness is a growing public health issue that causes visual discomfort, weariness, and visual problems that have a negative impact on the quality of life in the somatic, social, and mental sectors; everyday activities; and occupational productivity [15-18]. DES is linked to a diminished ability to do visually demanding activities such as reading, driving, and computer-related employment. The physical consequence of dry eye appears to be mostly connected with long-term pain, which causes continuous ocular surface irritation and, as a result, reduces the quality of life [19]. Given that DES is among the most prevalent concerns in the field of ophthalmology, we aim in this study to assess dry eye syndrome among contact lens users in the Jazan Region [13].

## Materials and methods

Study design and setting

This study employed a descriptive cross-sectional design targeting accessible adult contact lens users living in the Jazan Region in Saudi Arabia who accepted to participate in the study, as well as those who do not use contact lenses. The sample size for this study was determined using the Raosoft sample size calculator (Raosoft, Inc., Seattle, WA). Three hundred seventy-seven were required to obtain a 95% confidence interval and 5% margin of error using 50% as an accepted prevalence. A total of 367 participants who met the inclusion criteria completed the study survey, resulting in a participation rate of 95.5%. The study was approved by the Scientific Research Ethics Committee of Jazan University, with approval number REC-45/11/1138.

Data collection tool and process

Data were collected through a self-administered questionnaire adapted from a previously published study, which comprised four sections: sociodemographic data (age, a history of eye diseases, contact lens use, and the frequency of use), questions about dry eye syndrome (DES), the Contact Lens Dry Eye Questionnaire-8 (CLDEQ-8), and the Ocular Surface Disease Index (OSDI) [20]. Additionally, a pilot study was conducted on a sample of five male and five female participants to evaluate the face validity of the questionnaire, the clarity of the items used, and the completion time. The data collection instrument was changed to an electronic format to ease the distribution and recruitment of the targeted population. A web link was created and distributed through social media to get the necessary sample size. This study used a non-probability, convenient, nonrandom sampling method.

Statistical analysis

All statistical calculations were performed using IBM SPSS version 27.0.1 (IBM Corp., Armonk, NY). The analysis involved both descriptive and inferential statistics. Descriptive statistics were used to summarize and describe the study participants' characteristics and responses. Frequencies and percentages were calculated for categorical variables, such as the participants' responses to dry eye symptoms, the Contact Lens Dry Eye Questionnaire-8 (CLDEQ-8), and the Ocular Surface Disease Index (OSDI). Continuous variables, such as age, mean, standard deviations, median, and interquartile range, were calculated and tabulated. The Contact Lens Dry Eye Questionnaire-8 (CLDEQ-8) was used to assess the frequency and intensity of dry eye symptoms among contact lens users over the past two weeks. Each item was assigned a numerical value, with the total score reflecting the overall severity of the contact lens-related dry eye symptoms. Scores from the individual questions were summed to provide an overall score ranging from 0 to 37, with higher scores indicating more severe symptoms. The participants were then categorized according to their contact lens comfort status: contact lens discomfort (CLD), a score of ≥12 on the CLDEQ-8, and non-CLD, a score of <12 on the CLDEQ-8. The Ocular Surface Disease Index (OSDI) was used to evaluate the severity of dry eye symptoms and their impact on vision-related functions. Each item on the OSDI was scored on a five-point Likert scale, ranging from 0 (none of the time) to 4 (all of the time). To calculate the OSDI score, the scores for all 12 items were summed, and the total score was multiplied by 25 and divided by the number of questions answered. The final OSDI score ranges from 0 to 100, with higher scores indicating more severe symptoms. The OSDI scores were interpreted as follows: 0-12 indicated normal or no symptoms, 13-22 mild dry eye symptoms, 23-32 moderate dry eye symptoms, and 33-100 severe dry eye symptoms. To determine the association of various categorical variables with contact lens or CLD, the chi-square test and Fisher's exact test (where the expected count in cells was less than five) were employed, and to compare continuous variables such as age, the Mann-Whitney U test was applied. The nonparametric test was chosen owing to the non-normal distribution of the variable age assessed by the application of the Kolmogorov-Smirnov test (p < 0.05). The significance level for all statistical tests was set at p < 0.05, indicating a 95% confidence interval.

## Results

Our study included 367 participants as presented in Table 1, with an average age of 31.0 years; most were women (318, 86.6%) and highly educated (310, 84.5%), having undergraduate or postgraduate degrees. One hundred eighty-two (49.7%) of all the participants were diagnosed with myopia, while 74 (20.2%) had not been diagnosed with any eye disease. One hundred eighty-one (49.3) of the participants use contact lens frequently; 79 (43.6%) of them use it monthly, which means at least once a month. One hundred forty-six contact lens users (80.7%) reported practicing good hygiene by washing their hands before use and changing the lens solution after each use.

**Table 1:** Sociodemographic characteristics of the participants in the study (N = 367) N, frequency; %, percentage; SD, standard deviation

Sociodemographic characteristics		N	%
Age (mean ± SD)		31.0 ± 12.0	
Sex	Female	318	86.6%
	Male	49	13.4%
Educational level	Below secondary school	9	2.5%
	Secondary	48	13.1%
	Undergraduate or postgraduate	310	84.5%
Have you ever been diagnosed with an eye disease?	Farsightedness	70	19.1%
	Myopia	182	49.7%
	Others	40	10.9%
	None	74	20.2%
Are you a contact lens user?	No	186	50.7%
	Yes	181	49.3%
If you use contact lenses, how often do you use them?	Annually	42	23.2%
	Daily	30	16.6%
	Monthly	79	43.6%
	Weekly	30	16.6%
Do you wash your hands before using contact lenses and make sure to change the lens solution after each use?	No	9	5.0%
	Sometimes	26	14.4%
	Yes	146	80.7%

Table 2 presents the frequency of dry eye symptoms reported by the participants. For example, 109 (29.7%) sometimes felt that their eyes were dry, while 98 (26.7%) never experienced dryness. Gritty or sandy sensations were sometimes felt by 133 (36.2%) of the participants, and burning sensations were also reported by 125 (34.1%) sometimes, with 100 (27.3%) rarely experiencing it. A redness of the eyes was noted sometimes by 128 (34.9%), while 116 (31.6%) experienced it rarely. Crusting on lashes was rare, with 248 (67.6%) never experiencing it. Finally, 192 (52.3%) of the participants never had their eyes stuck shut in the morning, though 99 (27.0%) reported this symptom rarely. Overall, the data in Table 2 suggest that dry eye symptoms were not consistently severe across the participants.

**Table 2:** Dry eye symptoms N, frequency; %, percentage

Symptoms	Always		Mostly		Sometimes		Rarely		Never	
	N	%	N	%	N	%	N	%	N	%
During a typical day in the past two weeks, how often did your eyes feel dry?	30	8.17%	43	11.72%	109	29.70%	87	23.71%	98	26.70%
Do you ever feel a gritty or sandy sensation in your eyes?	23	6.27%	36	9.81%	133	36.24%	90	24.52%	85	23.16%
Do your eyes ever have a burning sensation?	26	7.08%	61	16.62%	125	34.06%	100	27.25%	55	14.99%
Are your eyes ever red?	17	4.63%	49	13.35%	128	34.88%	116	31.61%	57	15.53%
Do you notice crusting on your lashes?	12	3.27%	10	2.72%	41	11.17%	56	15.26%	248	67.57%
Do your eyes ever get stuck shut in the morning?	12	3.27%	14	3.81%	50	13.62%	99	26.98%	192	52.32%

**Figure 1:**
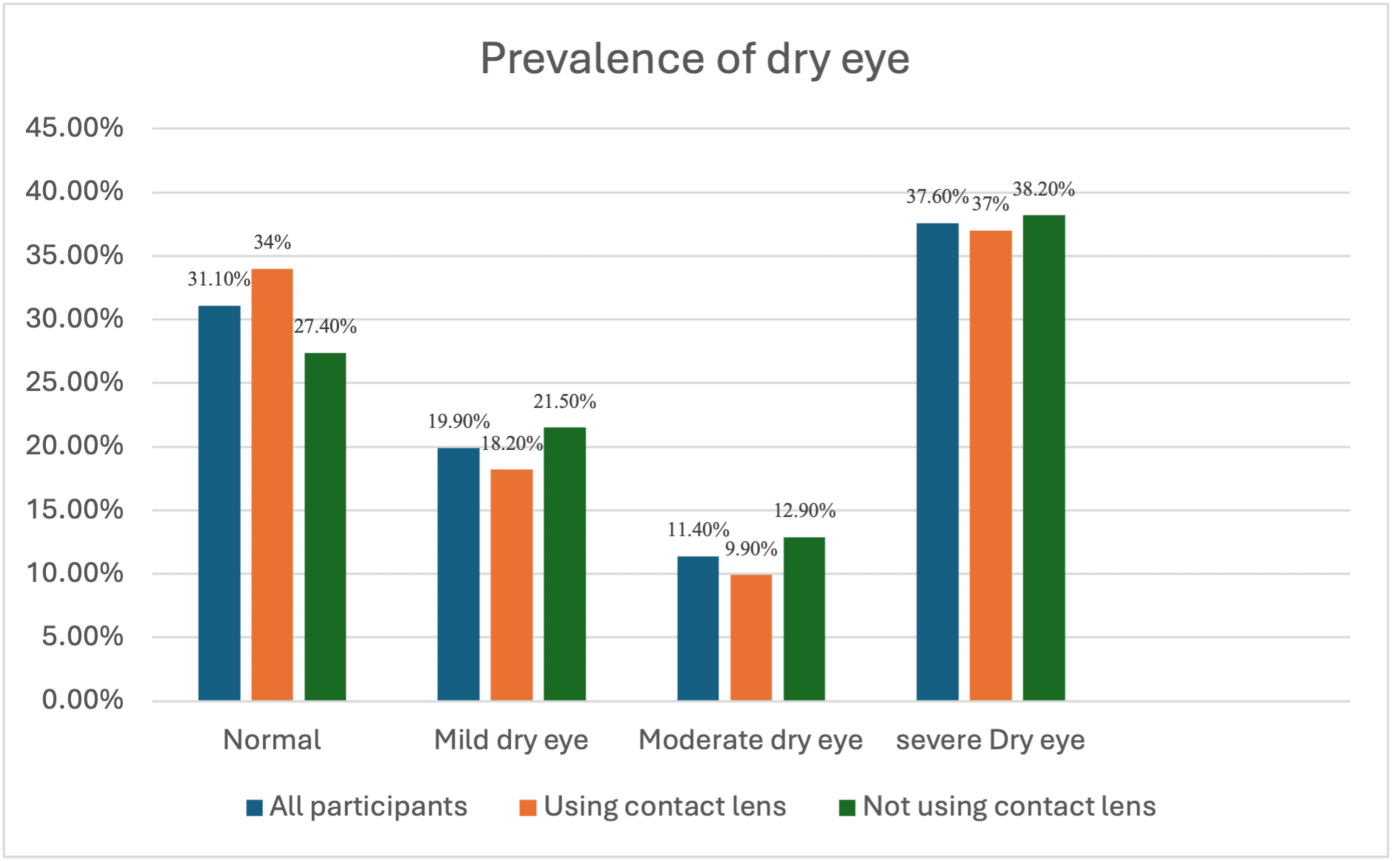
Prevalence of dry eye syndrome using OSDI OSDI: Ocular Surface Disease Index

Figure 1 shows the prevalence of dry eye by severity among contact lens users and non-contact lens users, as well as all the participants. It shows that 138 (37.6%) of all the participants experience severe dry eye and 114 (31.1%) of all the participants have no dry eye. However, 67 (37%) and 71 (38.2%) of contact lens users and non-contact lens users experience severe dry eye, respectively.

**Figure 2:**
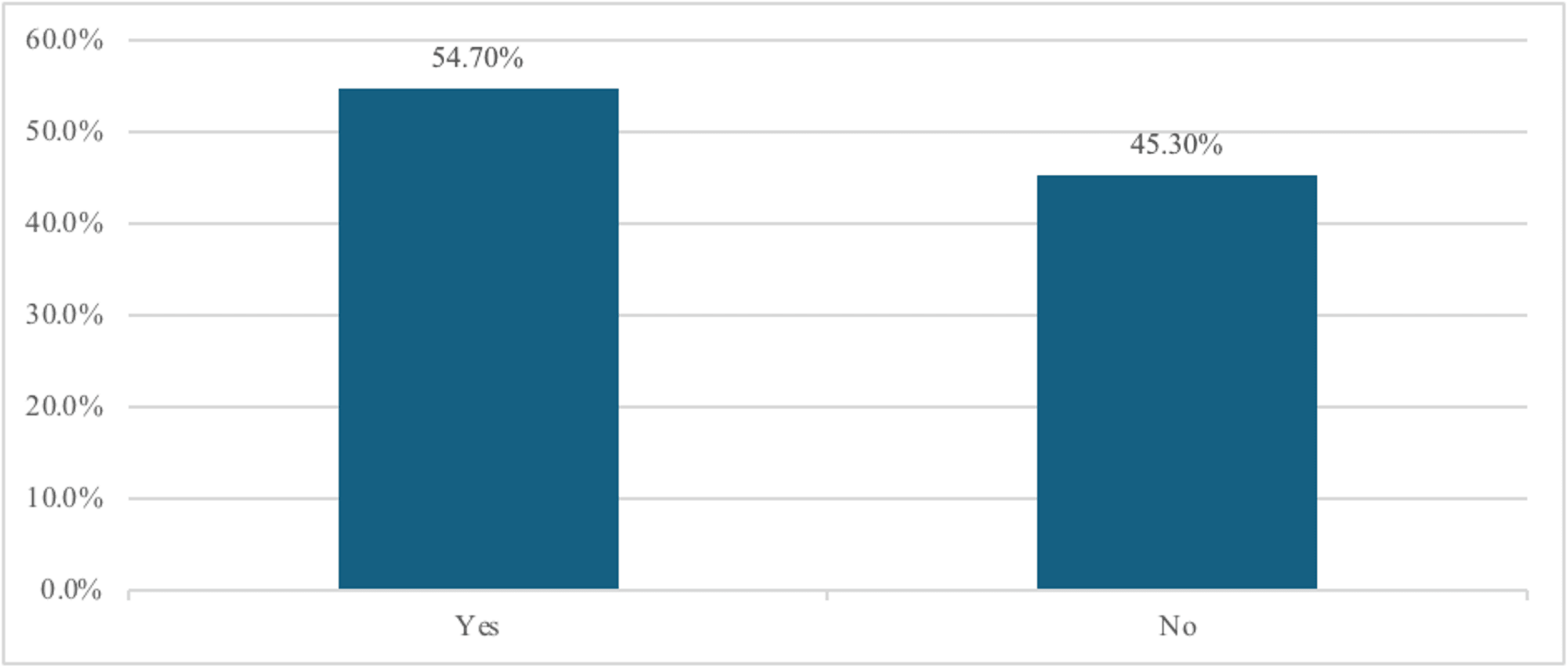
Contact lens-associated dry eye (CLADE)

Table 3 shows no statistically significant association between contact lens use and the severity of dry eye symptoms, with a p-value of 0.416. As shown in Figure 2, 99 (54.7%) of contact lens users reported having contact lens-associated dry eye (CLADE) using CLDEQ-8.

**Table 3:** Relation between the use of contact lenses and the incidence of dry eye ^C^Chi-square test N, frequency; %, percentage

Dry eye	Using contact lens				P-value
	No		Yes		
	N	%	N	%	
Normal	51	27.4%	63	34.8%	0.416^C^
Mild dry eye	40	21.5%	33	18.2%	
Moderate dry eye	24	12.9%	18	9.9%	
Severe dry eye	71	38.2%	67	37.0%	

Table 4 reveals that younger individuals are more likely to use contact lenses (p = 0.019). Gender also plays a significant role, as women are more likely to use contact lenses compared to men (p < 0.001). Although educational level shows a trend where higher education is associated with more contact lens use, it is not statistically significant (p = 0.055). Regarding eye disease diagnoses, those with myopia and other eye conditions are more likely to use contact lenses compared to those with farsightedness or no eye disease, with a significant p-value of less than 0.001.

**Table 4:** Association of sociodemographic data with the use of contact lens *P < 0.05, significant ^U^Mann-Whitney U test ^C^Chi-square test N, frequency; %, percentage; IQR, interquartile range

Sociodemographic data		Are you a contact lens user?				P-value
		No		Yes		
		N	%	N	%	
Age	Median (IQR)	26 (20-44)		24 (19-40)		0.019*^U^
Sex	Female	145	45.6%	173	54.4%	<0.001*^C^
	Male	41	83.7%	8	16.3%	
Have you ever been diagnosed with an eye disease?	Farsightedness	47	67.1%	23	32.9%	<0.001*^C^
	Myopia	75	41.2%	107	58.8%	
	No	46	62.2%	28	37.8%	
	Others	18	45.0%	22	55.0%	

Table 5 showed that CLD was significantly associated with age (p = 0.569). Gender shows no significant association (p = 1.000). The educational level also does not significantly impact CLD, with all groups having similar proportions (p = 0.812). Regarding eye disease diagnosis, those with myopia have a higher prevalence of CLD (66, 61.7%) compared to those with other diagnoses, though the difference is not statistically significant (p = 0.062). The frequency of contact lens use and its relation to CLD is not significant, with users across different usage frequencies reporting similar rates of CLD (p = 0.545). However, there is a significant association between CLD and hygiene practices: users who do not wash their hands before using lenses or change the lens solution after each use have a higher prevalence of CLD, with a p-value of 0.014.

**Table 5:** Distribution of contact lens-associated dry eye among lens users by their sociodemographic data *P < 0.05, significant ^U^Mann-Whitney U test ^C^Chi-square test ^F^Fisher's exact test N, frequency; %, percentage; CLD, contact lens discomfort; IQR, interquartile range

Sociodemographic factors		CLD				P-value
		Yes		No		
		N	%	N	%	
Age	Median (IQR)	24 (19-40)		24.5 (20-41)		0.569^U^
Sex	Female	95	54.9%	78	45.1%	1.000^F^
	Male	4	50.0%	4	50.0%	
Educational level	Below secondary school	1	100.0%	0	0.0%	0.812^F^
	Secondary	11	50.0%	11	50.0%	
	Undergraduate or postgraduate	87	55.1%	71	44.9%	
Have you ever been diagnosed with an eye disease?	Farsightedness	10	43.5%	13	56.5%	0.062^C^
	Myopia	66	61.7%	41	38.3%	
	No	10	35.7%	18	64.3%	
	Others	12	54.5%	10	45.5%	
If you use contact lenses, how often do you use them?	Annually	20	47.6%	22	52.4%	0.545^C^
	Daily or almost daily	17	56.7%	13	43.3%	
	Monthly	45	57.0%	34	43.0%	
	Weekly	17	56.7%	13	43.3%	
Do you wash your hands before using contact lenses and make sure to change the lens solution after each use?	No	9	100.0%	0	0.0%	0.014*^F^
	Sometimes	14	53.8%	12	46.2%	
	Yes	76	52.1%	70	47.9%	

## Discussion

The estimated prevalence of DES in specific cities in Saudi Arabia ranged from 32.1% to 75.9% [21-23]. Moreover, few studies have examined the potential relationship between contact lens use and the incidence and severity of DES. So, the purpose of this study is to explore dry eye syndrome among contact lens users, as well as non-contact lens users, in the Jazan Region. The study included 367 participants, 181 (49.3) of them were contact lens users, and 146 (80.7%) of them maintained adequate hygiene. Regarding dry eye symptoms, 109 (29.7%) reported occasional dryness, whereas 248 (67.6%) reported no crusting. The severity of dry eye varied; 138 (37.6%) of all the participants, including non-contact lens users, had severe dry eye. There was no statistically significant relationship between contact lens use and dry eye severity (p = 0.416). Ninety-nine (54.7%) of the respondents reported contact lens-associated dry eye (CLADE). Although those with myopia showed higher rates of CLD (66, 61.7%), this was not statistically significant. Poor hygiene practices were associated with higher CLD prevalence (p = 0.014).

In our study, we discovered that 181 (49.3) of the participants wore contact lenses, as shown in Table 1. This finding is similar to another national study conducted by Almutairi et al., which reported that almost 44% of the participants were using contract lenses [24]. In contrast, these findings were inconsistent with those found in Australia [25]. Most contact lens wearers in our study practiced appropriate hygiene, which opposed what was published in the Maldivian study, as they found that a major reported form of noncompliance was poor hand hygiene (60.7%) [26].

The frequency of dry eye symptoms was variable across all subjects; most symptoms only occurred infrequently or occasionally, as mentioned in Table 2. This is similar to what was reported in a previous study in the Asir Region [20] and discordant with what is observed in another study in Jeddah [27]. One hundred thirty-eight (37.6%) of all the subjects, including non-contact lens users, experienced severe dry eye. However, Almutairi et al. found that 57.4% of all the participants were having severe dry eye [24]. Moreover, the frequency of DES in Australia was estimated to be only 10.8% [25]. This wide range can be attributable to variances in environmental, genetic, and lifestyle variables.

In regard to the relationship between contact lens users and symptom severity, no significant association was noticed, and that was consistent with another local study published in 2021 [24]. Similarly, Helayel et al. found that contact lenses were not a significant risk factor for dry eye syndrome, perhaps due to their higher prevalence in our area due to other factors including dry and hot weather, which render the statistics for CL insignificant [28]. Moreover, severe dry eye affects 67 (37.0%) of contact lens users (Table 3), compared to the 4.5% reported severe dry eyes in a study conducted in the Asir Region [20].

Our findings indicated that the demographic groups with the highest prevalence of contact lens usage included younger individuals, women, and those with myopia (Table 4). This pattern closely resembles the results obtained from a study conducted by Alamri et al. [20]. Furthermore, numerous studies carried out in various regions of Saudi Arabia have consistently indicated that being a woman serves as an independent risk factor for the development of dry eye syndrome. This means that women may be more susceptible to experiencing this condition, regardless of other potential influencing factors [21,22,27]. Changes in the balance of sex hormones are thought to be the cause of this. The aqueous layer, lipid, and mucin are among the components of the tear film that are influenced by the sex hormones estrogens and androgens [29]. Although the participants in our study who had hyperopia and other eye diseases did not wear contact lenses in the same number as those with myopia, this observation aligns with the findings reported in the Asir study [20].

The CLDEQ-8 questionnaire revealed that 99 (54.7%) of the participants experienced dry eye related to contact lens use. However, it does highlight a significant correlation with hygiene practices, with individuals who maintain good hygiene being less likely to experience CLD. However, Alamri et al. found that 28% of contact lens users had significant CLD; moreover, no significant correlation was noticed with poor hygiene [20].

This was a cross-sectional observational study limited by the convenience sampling method, so it is expected to have the limitations associated with such a method. This discrepancy could stem from how the survey was distributed, potentially affecting the accuracy of the findings. Additionally, clinical diagnosis with tear film breakage tests is more reliable than questionnaires. However, the OSDI, utilized in this study, demonstrates a reliable technique for diagnosing DES [30].

## Conclusions

Although there was no significant correlation between the severity of dry eye and contact lens use, this study showed that dry eye symptoms were prevalent among contact lens users. However, a comparable prevalence was found among non-contact lens users as well. This finding could be explained by the other factors that influence the prevalence and severity of dry eye syndrome in the studied area, including dry and hot weather. This highlights the importance of raising ocular health awareness and education in this community. The data show that gender, myopia, and cleanliness behaviors all have a role in lens users' discomfort levels. Notably, women were more likely to wear contact lenses and reported more cases of dry eye problems. Furthermore, these findings argue for the creation of focused training programs that promote optimal practices in contact lens cleanliness and care, potentially improving user satisfaction. Future research should focus on longitudinal studies to evaluate the long-term impact of contact lens usage on ocular health, as well as other contributing factors across varied populations using clinical diagnosis with a tear film breakage test.

## References

[1] Gomes JA, Azar DT, Baudouin C (2017). TFOS DEWS II iatrogenic report. Ocul Surf.

[2] Tsubota K, Yokoi N, Shimazaki J (2017). New perspectives on dry eye definition and diagnosis: a consensus report by the Asia Dry Eye Society. Ocul Surf.

[3] Begley CG, Chalmers RL, Mitchell GL (2001). Characterization of ocular surface symptoms from optometric practices in North America. Cornea.

[4] Chalmers RL, Begley CG (2006). Dryness symptoms among an unselected clinical population with and without contact lens wear. Cont Lens Anterior Eye.

[5] Gipson IK (2004). Distribution of mucins at the ocular surface. Exp Eye Res.

[6] Doughty MJ (2011). Contact lens wear and the goblet cells of the human conjunctiva-a review. Cont Lens Anterior Eye.

[7] Riley C, Young G, Chalmers R (2006). Prevalence of ocular surface symptoms, signs, and uncomfortable hours of wear in contact lens wearers: the effect of refitting with daily-wear silicone hydrogel lenses (senofilcon a). Eye Contact Lens.

[8] Kastelan S, Lukenda A, Salopek-Rabatić J, Pavan J, Gotovac M (2013). Dry eye symptoms and signs in long-term contact lens wearers. Coll Antropol.

[9] Reddy SC, Ying KH, Theng LH, How OT, Fu-Xiang K, bin Mohamed Sikander MM (2016). A survey of dry eye symptoms in contact lens wearers and non-contact lens wearers among university students in Malaysia. J Clin Exp Ophthalmol.

[10] Chalmers RL, Young G, Kern J, Napier L, Hunt C (2016). Soft contact lens-related symptoms in North America and the United Kingdom. Optom Vis Sci.

[11] Lemp MA (1995). Report of the National Eye Institute/Industry workshop on clinical trials in dry eyes. CLAO J.

[12] Ranjan R, Shukla SK, Veer Singh C, Mishra BN, Sinha S, Sharma BD (2016). Prevalence of dry eye and its association with various risk factors in rural setup of western Uttar Pradesh in a tertiary care hospital. Open J Prev Med.

[13] Alharbi AJ, Alanazi NA, Alhamad JR, Alabdulqader RA, Aljamea DA, Alabdulqader SA, Naganathan M (2019). Prevalence of symptomatic dry eye and its risk factors among coastal population in Eastern Province of Saudi Arabia. EC Ophthalmol.

[14] Ibrahim NK, Seraj H, Khan R, Baabdullah M, Reda L (2018). Prevalence, habits and outcomes of using contact lenses among medical students. Pak J Med Sci.

[15] Smith JA (2007). The epidemiology of dry eye disease. Acta Ophthalmol Scand.

[16] Friedman NJ (2010). Impact of dry eye disease and treatment on quality of life. Curr Opin Ophthalmol.

[17] Uchino M, Schaumberg DA (2013). Dry eye disease: impact on quality of life and vision. Curr Ophthalmol Rep.

[18] Barabino S, Labetoulle M, Rolando M, Messmer EM (2016). Understanding symptoms and quality of life in patients with dry eye syndrome. Ocul Surf.

[19] Brown MM, Brown GC (2004). Utility assessment and dry eye disease. Ophthalmology.

[20] Alamri A, Amer KA, Aldosari AA, Al-Muhsin SD, Al-Maalwi RS, Al Hamdan SA, Al-Tarish LM (2022). Assessment of dry eye syndrome among contact lens users in Asir region, Saudi Arabia. Cureus.

[21] Alshamrani AA, Almousa AS, Almulhim AA (2017). Prevalence and risk factors of dry eye symptoms in a Saudi Arabian population. Middle East Afr J Ophthalmol.

[22] Alhamyani AH, Noor Kalakattawi RM, Noor Kalakattawi AM (2017). Prevalence of dry eye symptoms and its risk factors among patients of King Abdulaziz Specialist Hospital (Taif), Saudi Arabia. Saudi J Health Sci.

[23] Yasir ZH, Chauhan D, Khandekar R, Souru C, Varghese S (2019). Prevalence and determinants of dry eye disease among 40 years and older population of Riyadh (except capital), Saudi Arabia. Middle East Afr J Ophthalmol.

[24] Almutairi AH, Alalawi BS, Badr GH, Alawaz RA, Albarry M, Elbadawy HM (2021). Prevalence of dry eye syndrome in association with the use of contact lenses in Saudi Arabia. BMC Ophthalmol.

[25] Albietz JM (2000). Prevalence of dry eye subtypes in clinical optometry practice. Optom Vis Sci.

[26] Gyawali R, Nestha Mohamed F, Bist J, Kandel H, Marasini S, Khadka J (2014). Compliance and hygiene behaviour among soft contact lens wearers in the Maldives. Clin Exp Optom.

[27] Bukhari A, Ajlan R, Alsaggaf H (2009). Prevalence of dry eye in the normal population in Jeddah, Saudi Arabia. Orbit.

[28] Helayel HB, Al Abdulhadi HA, Aloqab A (2023). Prevalence and risk factors of dry eye disease among adults in Saudi Arabia. Saudi J Med Med Sci.

[29] Peck T, Olsakovsky L, Aggarwal S (2017). Dry eye syndrome in menopause and perimenopausal age group. J Midlife Health.

[30] Schiffman RM, Christianson MD, Jacobsen G, Hirsch JD, Reis BL (2000). Reliability and validity of the Ocular Surface Disease Index. Arch Ophthalmol.

